# Validation of a web-based self-administered test for cognitive assessment in a Swedish geriatric setting

**DOI:** 10.1371/journal.pone.0297575

**Published:** 2024-02-01

**Authors:** Einar Rystedt, Jakob Morén, Johan Lindbäck, Vitor Tedim Cruz, Martin Ingelsson, Lena Kilander, Nuno Lunet, Joana Pais, Luis Ruano, Gabriel Westman

**Affiliations:** 1 Department of Public Health and Caring Sciences, Geriatrics, Uppsala University, Uppsala, Sweden; 2 Department of Medical Sciences, Infection medicine, Uppsala University, Uppsala, Sweden; 3 Uppsala Clinical Research center, Uppsala University, Uppsala, Sweden; 4 Serviço de Neurologia, Unidade Local de Saúde de Matosinhos, Matosinhos, Portugal; 5 EPIUnit–Instituto de Saúde Pública, Universidade do Porto, Porto, Portugal; 6 Laboratório para a Investigação Integrativa e Translacional em Saúde Populacional (ITR), Porto, Portugal; 7 Krembil Brain Institute, University Health Network, Toronto, Ontario, Canada; 8 Departments of Medicine and Laboratory Medicine & Pathobiology, Tanz Centre for Research in Neurodegenerative Diseases, University of Toronto, Toronto, Ontario, Canada; 9 Departamento de Ciências da Saúde Pública e Forenses e Educação Médica, Faculdade de Medicina da Universidade do Porto, Porto, Portugal; 10 Serviço de Neurologia, Centro Hospitalar Entre Douro e Vouga, Santa Maria da Feira, Portugal; CNRS: Centre National de la Recherche Scientifique, FRANCE

## Abstract

Computerized cognitive tests have the potential to cost-effectively detect and monitor cognitive impairments and thereby facilitate treatment for these conditions. However, relatively few of these tests have been validated in a variety of populations. Brain on Track, a self-administered web-based test, has previously been shown to have a good ability to differentiate between healthy individuals and patients with cognitive impairment in Portuguese populations. The objective of this study was to validate the differential ability and evaluate the usability of Brain on Track in a Swedish memory clinic setting. Brain on Track was administered to 30 patients with mild cognitive impairment/mild dementia and 30 healthy controls, all scheduled to perform the test from home after one week and after three months. To evaluate the usability, the patient group was interviewed after completion of the testing phase. Patients scored lower than healthy controls at both the first (median score 42.4 vs 54.1, p<0.001) and the second test (median score 42.3 vs 55.0, p<0.001). The test-retest intra-class correlation was 0.87. A multiple logistic regression model accounting for effects of age, gender and education rendered an ability of Brain on Track to differentiate between the groups with an area under the receiver operation characteristics curve of 0.90 for the first and 0.88 for the second test. In the subjective evaluation, nine patients left positive comments, nine were negative whereas five left mixed comments regarding the test experience. Sixty percent of patients had received help from relatives to log on to the platform. In conclusion, Brain on Track performed well in differentiating healthy controls from patients with cognitive impairment and showed a high test-retest reliability, on par with results from previous studies. However, the substantial proportion of patients needing help to log in could to some extent limit an independent use of the platform.

## Introduction

With increasing age comes a higher risk for developing diseases that affect cognitive performance negatively. Consequently, due to the increasing life expectancy, cognitive impairment is a growing health problem worldwide. Early detection and diagnosis of cognitive impairment is crucial for giving adequate treatment and support to patients and caregivers [1]. Detection of disease as well as evaluation of treatments require neuropsychological assessments. Traditionally this has been accomplished by trained health care providers, such as neuropsychologists, physicians, or occupational therapists, administering paper and pen based cognitive tests. However, these tests are both time- and resource consuming, which may contribute to previous findings of dementia being underdiagnosed [2].

The progress in information technology during the last years has led to the development of computerized cognitive tests. Increasing access to, and experience with, computers among citizens of all ages have made these types of tests possible to administer to a large part of the population. Computerized, self-administered tests have the advantage that the execution requires less resources from the healthcare system and the tests can therefore be more widely and frequently distributed. Yet other advantages include a higher degree of standardization and that additional information, such as speed in answering, can be recorded easily. Tests that can be performed from home can also be more convenient and, particularly in rural areas, more accessible for patients. Several computerized tests have shown an ability to distinguish participants with mild cognitive impairment (MCI) or dementia from those that are cognitively unimpaired. However, a previous review has pointed out the need to have such tests validated in various populations. In particular, it is important to assess how they perform on individuals with different ethnicities, ages, educational backgrounds and causes of cognitive impairment [3,4].

Brain on Track (BoT) is an internet-based, self-administered cognitive test that can be performed from home. It has shown a high test-retest reliability and correlation with existing paper-pen tests [5]. Furthermore, a slightly revised version was able to discriminate between patients with MCI/mild dementia of varying causes and healthy controls in a southern European population with a low educational attainment as well as patients with multiple sclerosis and healthy controls of a median educational attainment [6,7].

The primary objective of this study was to evaluate the performance of BoT to accurately differentiate between patients with MCI/mild dementia and healthy controls in a Swedish Memory Clinic setting.

A secondary objective was to assess the usability of this type of self-administered internet based cognitive test in patients with MCI or mild dementia.

## Methods

### Study participants

For the validation, a target sample size of 60 was chosen (30 patients and 30 controls), in line with what is generally recommended in the literature for validation of an assessment test in a different language [8]. Participants with mild cognitive impairment or mild dementia were recruited among patients undergoing evaluation and treatment at the Memory Clinic at Uppsala University Hospital, Sweden, in line with a pre-defined set of inclusion criteria (Table 1).

**Table 1:** Inclusion criteria.

**All participants**	
• ≥18 years of age	
• No physical impairment precluding using a computer and mouse interface	
• Access to a computer at home	
• Being able to use a computer and mouse interface without external help	
**Patients**	**Controls**
Mild cognitive impairment	• Absence of any neurological, psychiatric or systemic disease that could impair cognition
• Subjective cognitive complaints over a period of at least 6 months	• Montreal Cognitive Assessment score above the cut point stratified by age and educational attainment (1.5 SD below mean)
• One or more cognitive domains ≥1.5 standard deviations (SD) below norm in standard cognitive tests	• Absence of drugs that could impair cognition in the past 3 months
• No clinical depression	• Absence of alcohol or substance abuse in the previous 2 years
• No impairment in daily activities	• No subjective memory complaints
*or*	
Mild dementia	
• Complying DSM-V criteria for major neurocognitive disorder	
• CDR score 0.5–1.0	

Mild cognitive impairment was defined as subjective cognitive complaints over a period of at least 6 months and at least one cognitive domain 1.5 standard deviation (SD) below the norm in standard cognitive tests, without impairment in daily activities and classified following Petersen criteria [9]. A diagnosis of dementia was made according to the criteria stipulated in Diagnostic and Statistical manual of Mental disorders–fifth edition (DSM-V) and the severity of dementia was classified using the Clinical Dementia Rating scale (CDR) [10,11]. The control group was recruited from healthy next of kin to patients seeking care at the memory clinic. After matching with the patient group for age and educational level, the subjects were approached for participation and included if meeting the inclusion criteria stratified in Table 1.

Recruitment continued until 30 participants in each group had been included and performed the first at-home test.

Data concerning age, gender, educational background, and computer usage was recorded for all participants.

### Cognitive testing

Brain on Track consists of eleven sub-tests measuring different aspects of cognitive ability (S1 Table). Each sub-test has a time limit which gives a total duration of approximately 24 minutes for the complete test. The sub-tests are presented in the same order in all tests but elements within the sub-tests are randomized (e.g. which words to remember) to minimize learning effects. Every correct answer within the time limit for each sub-tests gives 1 point. This raw score is transformed to a z-score by standardization using the mean and SD of the whole sample as the reference. The z-score for all sub-tests is then summarized and the sum again standardized using the mean and SD of the whole sample as the reference. The total score is achieved by multiplying this value by 10 and adding 50 to obtain a more intelligible score, so that most values are positive and vary from 0 to 100.

The patient interface language database for BoT was translated from English to Swedish, including welcome and end instructions, subtest instructions and lists of words used within the sub-tests. Spelling and grammar were reviewed and corrected through repeated testing of the Swedish version by multiple users, including reviewing a back-translation by a second bilingual translator. Audio versions of the instructions were created using an online text-to-speech tool [12].

After having given their informed consent all participants underwent a brief neuropsychological evaluation with the Montreal Cognitive Assessment (MoCA) version 7.1 and the Mini Mental State Exam (MMSE) and were given a brief training session on the BoT system [13,14]. In this training session, participants were shown how to log in to the platform and performed three sub-tests (Visual Memory Task II, Opposite Task and Color Interference Task) under supervision. All participants were recruited and evaluated by the same member of the research team.

After the initial evaluation the participants were instructed to perform the complete BoT test, including the previously performed sub-tests, from home twice–after one week and after three months. If participants were unable to keep the time plan, the reason for that was recorded. If a scheduled test time was missed a reminder was sent out twice. An additional reminder was sent out if a reason for missing the test (e.g., medical circumstances, computer malfunctions) was given.

For the assessment of the patient experience and usability of the test, all patients were interviewed using a structured questionnaire in Swedish (S2 Table) after completing the study, covering technical issues and general experience.

### Statistical analysis

Differences in BoT test scores between groups were analyzed with the Wilcoxon-Mann-Whitney test. Effect sizes including confidence interval were analyzed using Welch two sided t-test. The association between the BoT, MMSE, and MoCA scores were assessed by calculating the Spearman correlation coefficient (rho). The relation between BoT and the other two scores were presented graphically as a scatterplot with a locally estimated scatterplot smoother (loess) added to illustrate the relationship.

A multivariable linear regression analysis was used to assess the association between the BoT score as dependent variable and age, sex and education as independent variables. To assess differential effects between patients and controls, a model including pairwise interactions between the two groups and each of the other variables were included. The predictive accuracy of the test and the areas under the corresponding receiver operating characteristic (ROC) curves was calculated using multivariable logistic regression models.

Test-retest reliability was assessed using consistency two way mixed single intraclass correlation coefficient (ICC) [15]. The ICC was estimated using a linear mixed effects model.

Statistical analyses were performed in R version 4.1.2 [16].

### Ethical considerations

Ethics approval was obtained from the Regional ethics committee in Uppsala, Sweden. Written informed consent was obtained from all participants.

## Results

A total of 37 patients with MCI/mild dementia were included and completed the training session in BoT. Of these, 30 patients proceeded as instructed to perform the first at-home test that had been scheduled one week later. The reasons for not continuing study participation were that the participant: found the test too hard (n = 2), forgot to take the test (n = 1), or was not able to use the computer (n = 1). Three patients did not state any reason for discontinuation.

Similarly, 39 subjectively cognitively healthy individuals were included. Five participants performed below the stratified cut-off score on MoCA and were therefore excluded [17]. One participant felt stressed after completing MoCA and did not wish to participate further. The remaining 33 participants were included as healthy controls. Of these, two did not perform BoT from home and did not respond to reminders, and one lost access to internet and was therefore unable to participate. The remaining 30 healthy controls performed BoT from home after one week.

**Fig 1:**
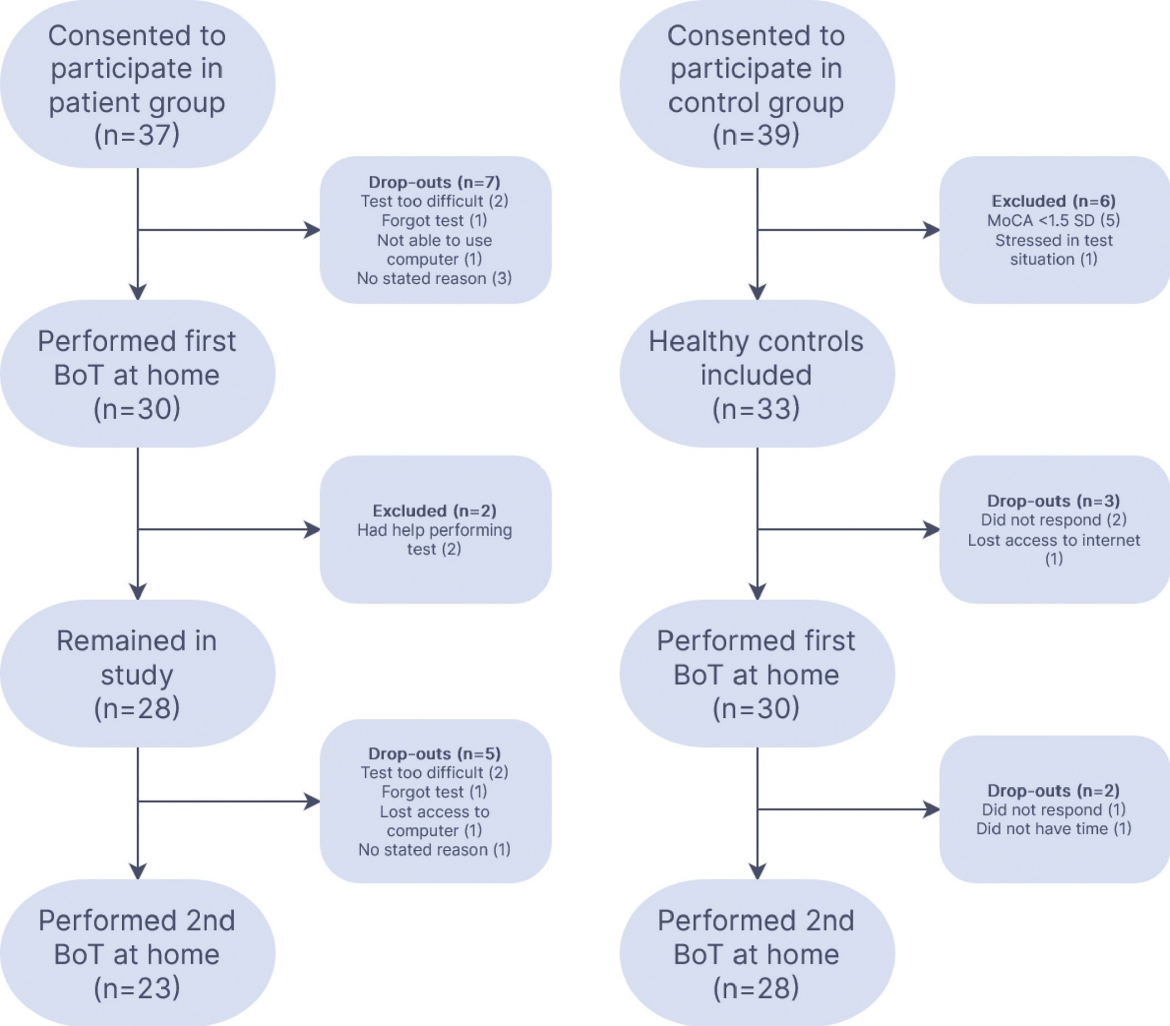
Overview of study participation.

The second at-home test, scheduled three months from inclusion, was completed by 25 patients and 28 controls. The reasons for missing the second test were similar to the reasons for missing the first test. In the patient group, one did not want to do the test for unspecified reasons, two thought it was too hard, one forgot and one lost computer access. In the control group one did not respond to the maximum number of reminders given (2), and one did not have time to participate further. At follow up it was revealed that two patients received help performing the test and were therefore excluded (Fig 1).

Of the 28 participants remaining in the patient group, 17 had mild cognitive impairment (13 of amnestic and 4 of multidomain type) and 11 mild dementia. In seven cases, dementia was due to Alzheimer’s disease and in one case each: dementia with Lewy bodies, frontotemporal dementia, Parkinson’s disease and mixed Alzheimer’s disease and vascular dementia.

Patients that after inclusion did not perform any BoT test from home had a lower test score in MMSE (median 20.0 vs 24.5) and MoCA (median 16.0 vs 20.5) than the remaining patients (p<0.01). Of the seven patients not participating in BoT from home three (43%) had a diagnosis of MCI and four (57%) a diagnosis of mild dementia.

The MCI/dementia patients had a lower score than the healthy controls on the BoT test at both the first test at home after one week (median score 42.4 vs 54.1, p <0.001) and at the second test after three months (median score 42.3 vs 55.0, p <0.001) (Table 2).

**Table 2:** Participants’ demographics.

	**Controls**	**MCI or dementia**	**P-value**
**Age**	72 (54–80)	74 (52–81)	
**Women**	50%	43%	
**Education** **(years)**	15.8 (7–22.5)	14.5 (7–22)	
**Daily computer** **usage**	83%	61%	
**MMSE score**	29 (25–30)	24.5 (21–29)	<0.001
**MoCA score**	28 (25–30)	20.5 (13–24)	<0.001
**BoT score** **first session**	54.1 (35.9–79.7)	42.4 (32.5–58.6)	<0.001
**BoT score** **second session**	55.0 (43.1–78.1)	42.3 (32.6–56.4)	<0.001
	Controls (mean value)	MCI or dementia (mean value)	Difference (95% CI)
**MMSE score**	28.5	24.6	3.9 (2.9, 4.9)
**MoCA score**	28.1	20.8	7.4 (6.3, 8.4)
**BoT score** **first session**	55.6	43.0	12.6 (8.5, 16.7)
**BoT score** **second session**	56.3	43.7	12.6 (8.3, 17.0)

MMSE and MoCA performed at inclusion, BoT from home after one week and three months. Numbers are median (max-min) unless otherwise stated. Differences in test scores between groups were analyzed with the Wilcoxon-Mann-Whitney test. Effect sizes with 95% confidence interval were analyzed using Welch two sided t-test.

Participants with mild dementia had a statistically non-significant tendency towards lower scores on all cognitive tests compared to participants with MCI. Median MMSE score was 23.0 vs 25.0, MoCA 20.0 vs 21.0 and first and second BoT test 42.3 vs 43.4 and 38.5 vs 45.0 for the participants with dementia and MCI, respectively.

**Fig 2:**
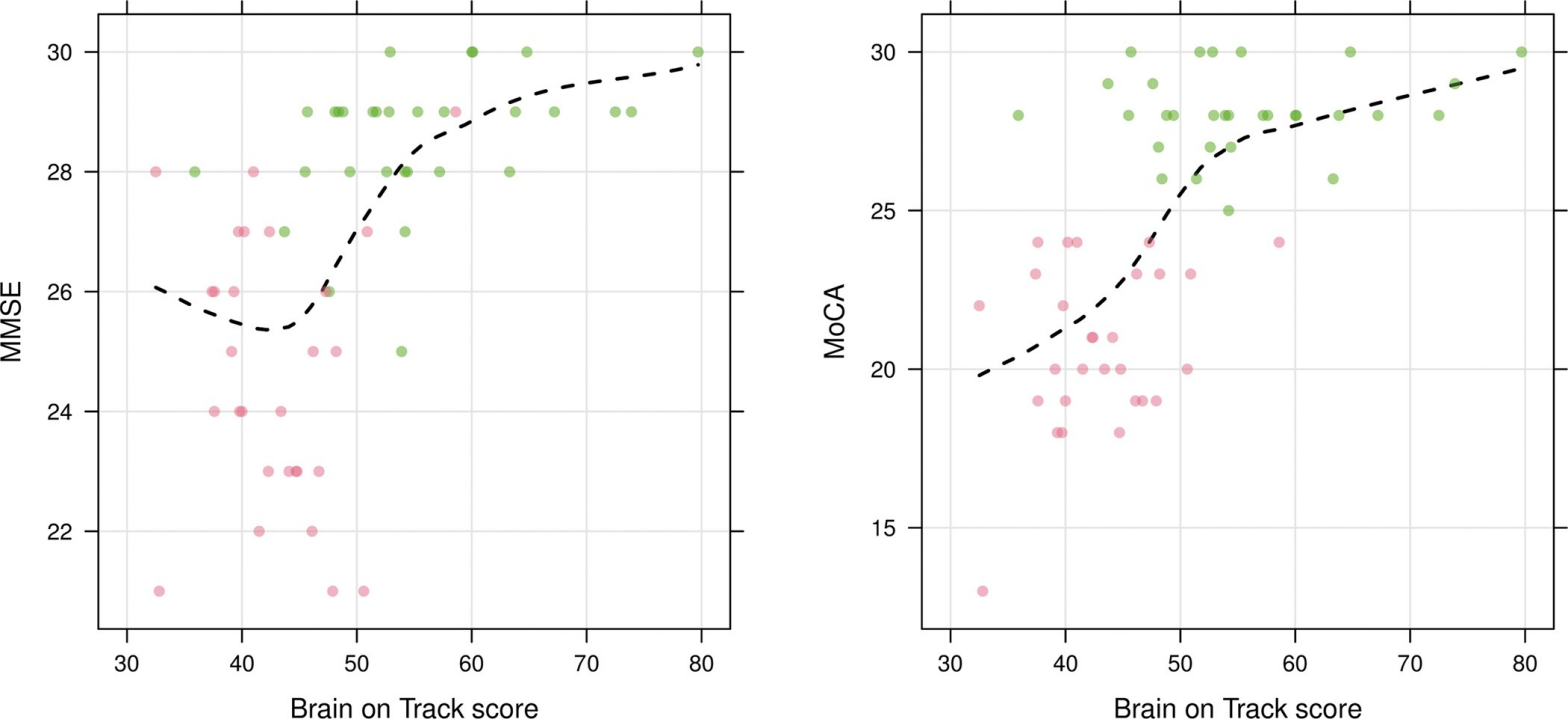
Test score correlations. Test score on MMSE and MoCA respectively with corresponding score on first BoT test from home. Controls colored green and patients red. Rho between MMSE and BoT; 0.64 (p<0.001), between MoCA and BoT; 0.64 (p<0.001). Estimated scatterplot smoother (loess) added to illustrate the relationship.

The BoT test score was positively correlated with the score on MMSE (rho 0.64, p<0.001) and MoCA (rho 0.64, p<0.001) (Fig 2). Correlation matrix for the subtests of MoCA and BoT are shown in the supplement (S3 Table).

**Fig 3:**
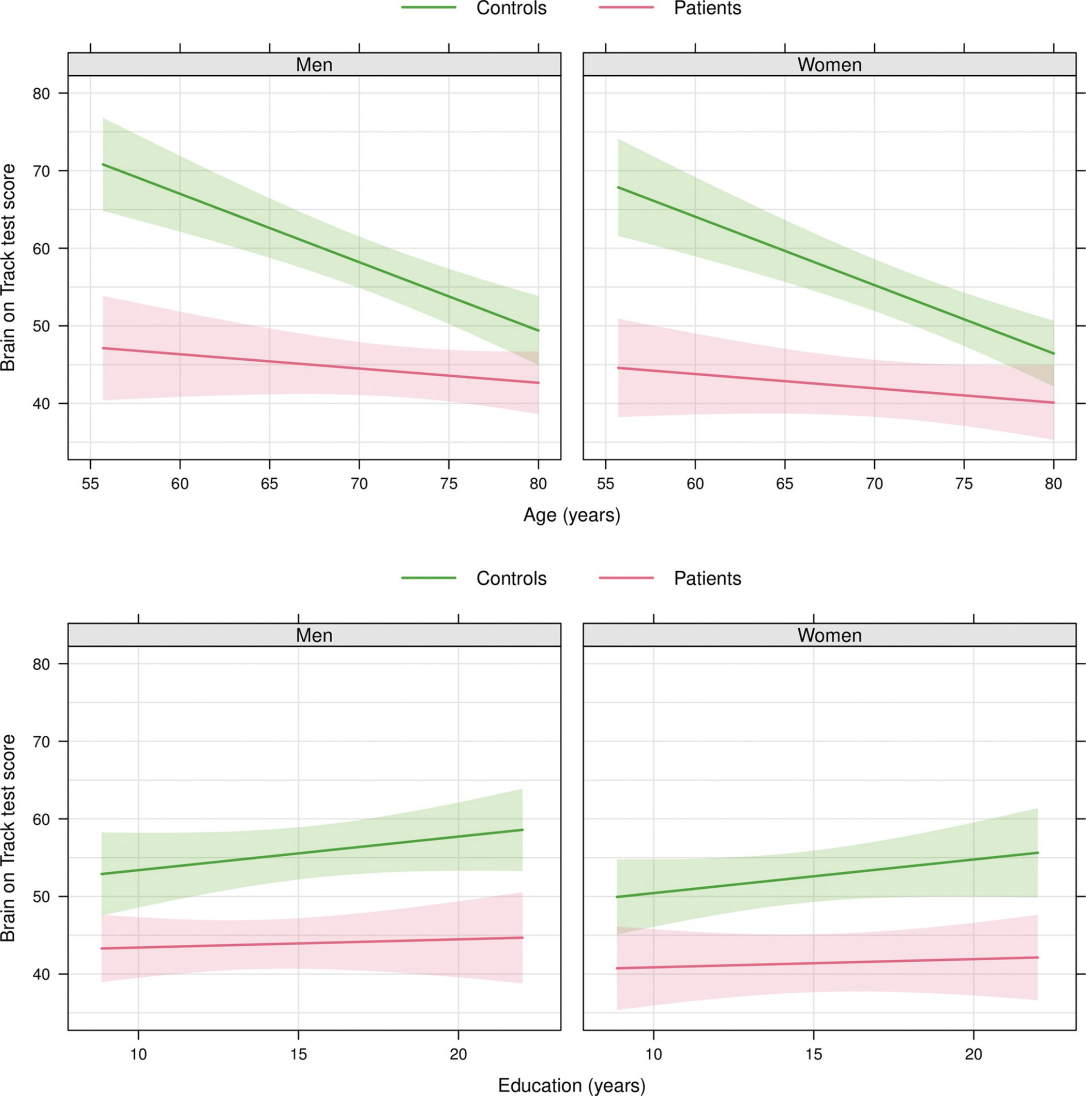
Associations between Brain on Track score and age (top) and education (bottom) by sex and group. The associations are predictions from a single multiple linear regression model including group, age, sex, education, as well as interactions between group and age, group and sex, and group and education. Each variable included in the model needs to be assigned at least one value. Therefore, the associations in the top panels are plotted for an education level of 15 years and in the bottom panels for an age of 73 years. The only interaction that was statistically significant was between group and age (p = 0.005).

The score on MMSE was positively correlated with the score on MoCA (rho 0.80, p<0.001). The association between BoT and age was stronger among the controls than in MCI/dementia patients (p for interaction = 0.005, and for overall association < 0.001, Fig 3), with a larger difference between controls and patients among the younger than among the older. The overall associations with education (p = 0.37) and gender (p = 0.27) were not statistically significant and there were no indication of any differential effects between the groups (p for interaction 0.45 and 0.91, respectively).

BoT showed an ability to differentiate patients from healthy controls with an area under the ROC curve of 0.90 and 0.88 for the first and second test respectively (S1 Fig).

**Fig 4:**
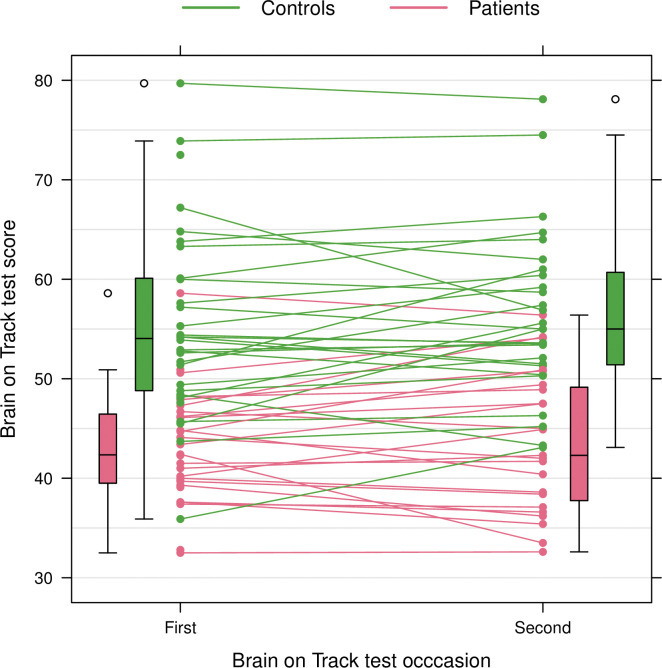
Brain on Track test scores at two occasions for patients (red) and controls (green). The box plots show the distributions of the respective test scores at the two occasions.

The test-retest intra-class correlation (ICC) was 0.87 (Fig 4). The ICC was estimated using a linear mixed effects model including patient/control group as a fixed effect to account for the study design. This essentially makes the ICC a weighted average of the within-group ICCs. The difference in median score between the first and second BoT test was 0.9 vs -0.1 for the patient and control groups respectively (n.s).

The ICC for the subtest “Delayed verbal memory” was 0.01, the ICC for the remaining subtests varied between 0.46–0.81 (Table 3).

**Table 3:** The ICC for each BoT subtest.

Subtest	ICC	Variance between subjects	Variance within subjects
Attention task III	0.49	0.39	0.41
Visual memory task II	0.46	0.32	0.38
Delayed verbal memory task	0.01	0.005	0.57
Calculus task	0.49	0.34	0.36
Color interference task	0.49	0.36	0.37
Verbal memory task II	0.59	0.53	0.36
Opposite task	0.81	0.68	0.16
Written comprehension	0.77	0.64	0.19
Word categories	0.66	0.50	0.25
Sequences	0.76	0.58	0.18
Puzzles	0.73	0.63	0.24

ICC = Variance between subjects within groups / (variance between subjects within groups + variance within subjects).

Of the 28 patients included in the analyses, 26 had participated in the follow-up. Fifteen of the responders had received assistance from a relative when logging on to the BoT platform, but not with the actual test, ten responders managed without any support, and one responder did not remember whether he/she needed any assistance. Thirty-two percent of the patients found BoT to be an easy test to perform while 23% found it difficult. Regarding free comments there were an equal number of participants giving only negative (n = 9) or only positive (n = 9) feedback and five giving mixed comments. Comparing participants giving only positive comments with participants giving only negative comments, the latter were more likely to have a diagnosis of dementia (56 vs 11%) and less likely to perform the second test from home (67 vs 100%). They also had a lower score on BoT, median score 40.2 vs 46.1 and 41.6 vs 47.5 for the first and second test respectively. The most common negative comments were not appreciating the time limit for each subtest (50%) followed by a sense of stress in the test situation (29%) and difficulties handling the computer (14%). The most common positive comments were that the test felt interesting or stimulating (57%) followed by a positive feeling about doing the test from home instead of at the clinic (21%).

## Discussion

This study indicates a good predictive ability of the BoT test to detect MCI or mild dementia in a Swedish population with a high educational attainment. The diagnostic accuracy with an AUC of 0.90 for single use was in par with the accuracy seen in previous studies [6,7]. Further, BoT correlated well with the widely used standard neuropsychological tests Montreal Cognitive Assessment and Mini Mental State Examination although not as strongly as these tests correlated with each other. This was not surprising given the significant similarities between MMSE and MoCA in deliverance, configuration and scoring. As is the case with traditional paper and pen tests there is nowadays a wide variety of different computerized tests developed for use in different clinical situations, e.g. the THINC-it tool used to screen for cognitive dysfunction in depression [18]. Several short screening tests for individuals at risk for dementia have been integrated into clinical practice, e.g. National Institutes of Health Toolbox Cognition Battery and CogState [19,20]. Nevertheless, despite their potential benefits in replacing conventional pen-and-paper screening tests, these assessments still necessitate the presence of a trained evaluator and a clinic visit by the patient.

Comparing self-administered computerized tests, some others have reported an AUC between 0.82–0.91 in differentiating healthy controls from participants with MCI/mild dementia [21–23]. A direct comparison between the different tests predictive abilities is however difficult to make due to differences in studied populations and included participants. In a Swedish memory clinic setting, the self-administered Geras Solutions Cognitive Test showed a discriminatory ability between participants with only subjective cognitive complaints and those with MCI/dementia of 0.80 [24]. For comparison the established paper and pen test Montreal Cognitive Assessment has in previous studies showed a weighted discriminatory ability between healthy controls and mild cognitive impairment of 0.89 [25].

The test-retest reliability of BoT appears to be high, with an intra-class correlation of 0.87. The ICC for individual subtests were lower, which is not unexpected, with Delayed verbal memory task lower than other subtests with an ICC of only 0.01 caused by a low variance in score within each group for this subtest. Given the small sample size it is difficult to interpret these results but it constitutes an interesting finding that is worth studying further. The overall high ICC indicates that random effects and potential test learning factors are not of substantial size compared to the difference between patients and control. Together with findings from previous studies [6,7] these results indicate that BoT may serve as a reliable tool for measuring cognitive performance in populations with a variety of educational- and cultural backgrounds. For comparison, the established MoCA has showed an ICC between version 7.1 and 7.2 of 0.64 and between version 7.1 and 7.3 of 0.82 [26]. It is however precarious to make direct comparison of the exact values as differences in e.g. studied populations affects these.

It is interesting to note that the learning effects were less evident in this sample of Swedish participants than in the Portuguese studies, performed in a population that has much lower levels of educational attainment [6,7]. This suggests that learning effects could become increasingly less relevant as more educated cohorts reach older ages.

It is worth noting the composition of the patient group, presenting with relatively mild cognitive problems and with a mean score of 25 on MMSE which is just above the commonly used cut-off of ≤24 which is considered indicative of cognitive disorder. The group contains a large proportion with amnestic MCI or mild Alzheimer’s disease. This is in line with the most common patient group seeking care at a memory clinic where a possible implementation of web-based cognitive testing could be considered and suggests that the BoT test is relevant in a common clinical situation.

A cognitive test with a good ability to detect and distinguish individuals with incipient cognitive difficulties, which can be administered at low costs to a large group of people has the potential to significantly improve care. In addition to delivering reliable and meaningful results, such a test must also have a certain degree of ease of use to be applicable in practice. The participants from the patient group indicated mixed experiences of completing the BoT test where, above all, the degree of difficulty and perceived stress of the time limit were raised as negative experiences and reasons for not completing the participation. However, an equally large group mentioned positive experiences, mainly an experience that it was interesting and stimulating, followed by the convenience of being able to perform cognitive testing from home. This is in line with the results from a previous qualitative study of patients’ experience of a computerized cognitive test [27]. There seems to be a correlation to more severe disease and/or lower test score and experiencing the test negatively as well as being more likely not to perform the test. This is an interesting finding that could point to difficulties in evaluating and following patients with BoT in more severe stages of cognitive dysfunction.

A potential problem was the relatively large proportion of study participants that did not complete the entire study, as only 23 of 37 patients (62%) completed both tests from home correctly. However, this should be seen in the context of the fact that the patient group consisted of individuals with known cognitive difficulties in everyday life and in customary neuropsychological tests and that participation in this study did not entail any benefit in treatment or financial compensation, while self-perceived difficulties in tests are often experienced negatively by patients. A large proportion of the patient group (60% of those who answered the question) also had help from relatives in logging into the platform, which could limit usability in real-life settings. In a computer based cognitive test performed without supervision there are some factors that are difficult to take into account that could have influenced participants test results such as motivation to perform the test, technological skills and interference from relatives in the test situation.

Looking ahead, the proportion of the population that could be considered for web-based cognitive testing will likely increase in the future as the experience in using computers, hand-held devices and digital user interfaces increases in the population.

## Supporting information

S1 Fig Receiver operation characteristic curves for the first and second Brain on Track test. (TIF)

S1 Table Description of Brain on Track subtests. (DOCX)

S2 Table BoT subjective evaluation. Translated from Swedish original.
(DOCX)

S3 Table Correlation matrix for BoT and MoCA subtests. (DOCX)

S4 Table Individual test scores. (DOCX)

S5 Table Raw score. Number of correct answers for each BoT subtest.
(DOCX)
