# Supplementary figures and images for: Validation of a web-based self-administered test for cognitive assessment in a Swedish geriatric setting
Source: PLoS One. 2024 Feb 1;19(2):e0297575. doi: 10.1371/journal.pone.0297575 (PMC10833583; doi:10.1371/journal.pone.0297575)

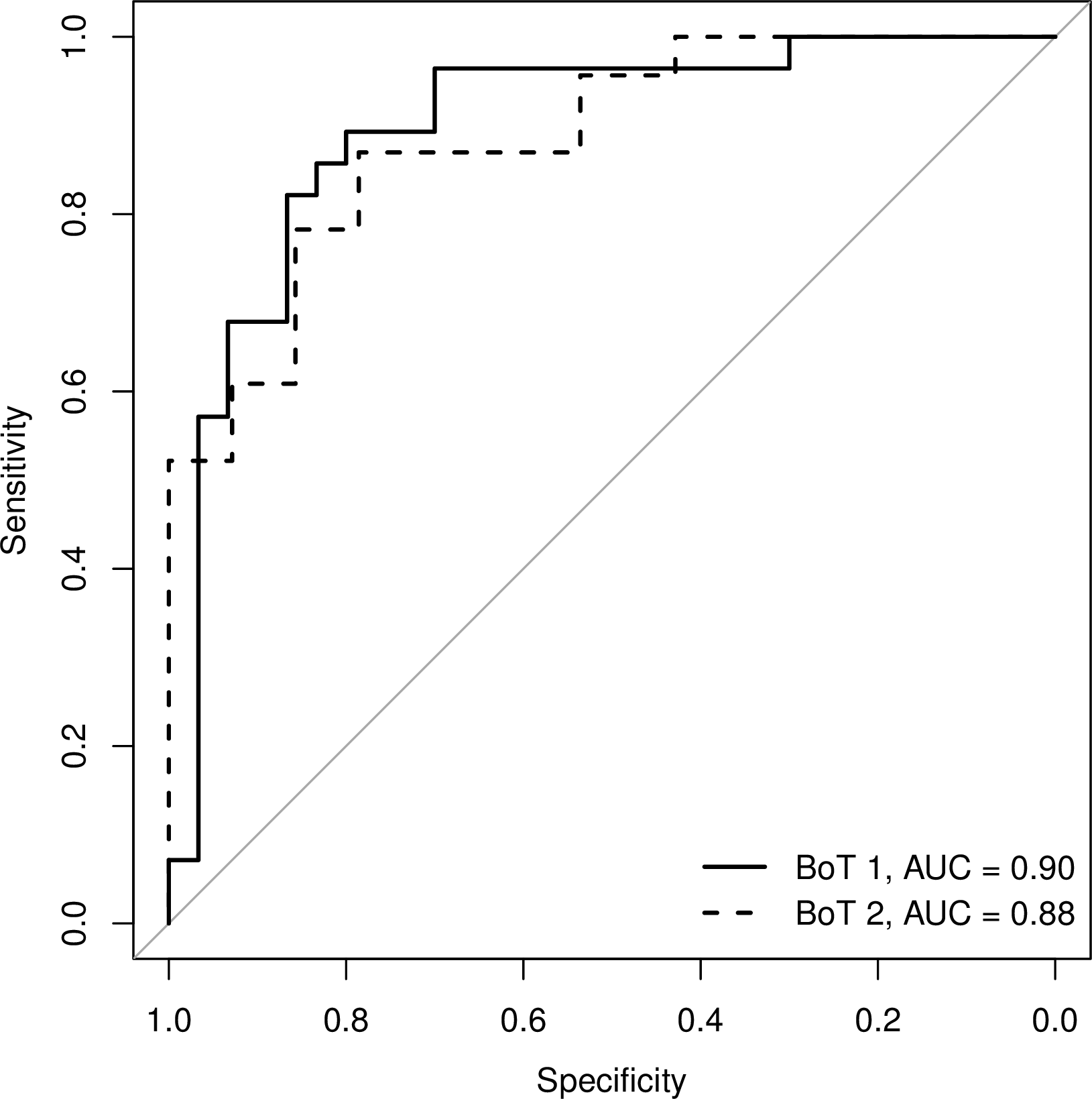

Supplement: S1 Fig — (TIF) [file pone.0297575.s001.tif]
