# Supplementary material for: Validation of a web-based self-administered test for cognitive assessment in a Swedish geriatric setting
Source: PLoS One. 2024 Feb 1;19(2):e0297575. doi: 10.1371/journal.pone.0297575 (PMC10833583; doi:10.1371/journal.pone.0297575)
Supplement: S1 Table — (DOCX) [file pone.0297575.s002.docx]

**S1 Table. Description of Brain on Track subtests.**

| Name | Target Domains | Subtest Description |
| --- | --- | --- |
| Puzzles | Constructive ability | The screen is divided into two parts: on the right the target image is shown, on the left several of the image composing pieces are scattered. The purpose of the task is to complete the image using the scattered pieces. |
| Opposite Task | Inhibitory control  Executive functioning | In a central position of the screen, a large arrow is shown. The participant must press the keyboard arrow in the opposite direction to that shown by the large arrow. |
| Visual Memory Task II | Attention  Short term memory | On the screen, three cubes of different colors light up in a random sequence. The participant must memorize this sequence and reproduce it using the mouse to click on the cubes in the correct order. |
| Calculus Task | Calculus | The participant should perform the numerical calculation shown on screen and input the number via keyboard or by using the mouse to click on a keypad with numbers on screen. The operation should be completed before a balloon reaches the top of the screen. |
| Sequences | Executive function Abstract thought | The upper part of the screen displays a set of figures that follows a certain logic sequence. The participant should select the figure that completes the sequence from four possible figures. |
| Verbal Memory Task II | Immediate verbal memory | The participant is asked to memorize a list of three words. After a short delay, six words are shown on screen (three correct and three distracters) and the participant must click on the correct words. |
| Written Comprehension | Language comprehension | On the screen, there are several sets of geometric objects of different shapes and colors. The participant must select the set that matches the description of the written command. |
| Word categories | Language | The participant must select the correct category for the word that is shown on the screen by dragging the word to the corresponding box. If the word does not belong to any of the categories, the participant must drag it to the garbage can. |
| Color Interference task | Executive function, inhibitory control | A name of a color is shown inside a colored frame. The color name, the color of the word font and the color of the frame are random. In the first set, the participant must select YES when the word and the color of the frame match, and NO when they are different. In the middle of the subtest, a new instruction appears on screen, and now the participant must select YES when the color of the frame matches the color of the word font. |
| Delayed Verbal Memory Task | Short term memory | The participant is asked to memorize a list of five. After this, the participant is asked to recall the words after 90 seconds, after 180 seconds and after 360 seconds, performing an interference task between recalls. In the recall, four, six or 10 words, respectively for each level, are shown on screen. Half the words on screen are correct and half are distracters, the participant must click on the correct words. The words are randomly selected from a list of 50 words for each level, with increasing complexity. |
| Attention task III | Attention, information processing speed | Two pictures are shown on screen, each picture is composed of 15 geometrical shapes of different colors. The participant must decide if the two pictures are equal or different. The shapes and colors are randomized for each trial. |
