# Supplementary material for: Validation of a web-based self-administered test for cognitive assessment in a Swedish geriatric setting
Source: PLoS One. 2024 Feb 1;19(2):e0297575. doi: 10.1371/journal.pone.0297575 (PMC10833583; doi:10.1371/journal.pone.0297575)
Supplement: S2 Table — Translated from Swedish original. (DOCX) [file pone.0297575.s003.docx]

**S2 Table. BoT subjective evaluation.** Translated from Swedish original.

1. Did you receive any help logging in to the system? (Yes/No)
2. Did you receive any help with the test itself? (Yes/No)
3. If so, what test and what help? (Open text input)
4. Was it easy to log in to the system? (Yes/Fairly/No)
5. Was the test itself easy? (Yes/Fairly/No)
6. How did you experience performing the test? (Open text input)
7. Were any subtests more difficult? If so – which one? (Open text input)
8. Other comments (Open text input)
