# Supplementary material for: Validation of a web-based self-administered test for cognitive assessment in a Swedish geriatric setting
Source: PLoS One. 2024 Feb 1;19(2):e0297575. doi: 10.1371/journal.pone.0297575 (PMC10833583; doi:10.1371/journal.pone.0297575)
Supplement: S3 Table — (DOCX) [file pone.0297575.s004.docx]

**S3 table. Correlation matrix for BoT and MoCA subtests.**

|  | **Visuospatial**  **/Executive** | **Naming** | **Attention** | **Language** | **Abstraction** | **Delayed recall** | **Orientation** |
| --- | --- | --- | --- | --- | --- | --- | --- |
| **Attention task III** | **0.20** | **-0.12** | **0.07** | **0.25** | **0.20** | **0.32** | **-0.03** |
| **Visual memory task II** | **0.37** | **-0.01** | **-0.04** | **0.16** | **0.19** | **0.48** | **0.42** |
| **Delayed verbal memory task** | **0.53** | **0.09** | **0.01** | **0.42** | **0.25** | **0.56** | **0.44** |
| **Calculus task** | **0.51** | **0.26** | **0.16** | **0.39** | **0.32** | **0.36** | **0.15** |
| **Color interference task** | **0.36** | **0.18** | **0.01** | **0.23** | **0.33** | **0.44** | **0.19** |
| **Verbal memory task II** | **0.25** | **0.18** | **0.05** | **0.34** | **0.23** | **0.09** | **0.04** |
| **Opposite task** | **0.53** | **0.02** | **0.14** | **0.41** | **0.29** | **0.33** | **0.23** |
| **Written comprehension** | **0.37** | **0.15** | **0.30** | **0.38** | **0.36** | **0.33** | **0.16** |
| **Word categories** | **0.48** | **0.01** | **0.02** | **0.41** | **0.37** | **0.45** | **0.27** |
| **Sequences** | **0.25** | **-0.11** | **0.09** | **0.20** | **0.14** | **0.36** | **0.24** |
| **Puzzles** | **0.39** | **-0.06** | **0.16** | **0.26** | **0.30** | **0.46** | **0.06** |
