# Supplementary material for: Validation of a web-based self-administered test for cognitive assessment in a Swedish geriatric setting
Source: PLoS One. 2024 Feb 1;19(2):e0297575. doi: 10.1371/journal.pone.0297575 (PMC10833583; doi:10.1371/journal.pone.0297575)
Supplement: S4 Table — (DOCX) [file pone.0297575.s005.docx]

**S4 Table. Individual test scores.**

| **Group** | **BoT test 1** | **BoT test 2** | **MMSE** | **MoCA** |
| --- | --- | --- | --- | --- |
| **Control** | **54.2** | **51.5** | **28** | **28** |
| **Control** | **51.4** | **61.0** | **29** | **26** |
| **Control** | **54.2** | **53.6** | **27** | **25** |
| **Control** | **72.5** |  | **29** | **28** |
| **Control** | **45.7** | **46.3** | **29** | **30** |
| **Control** | **55.3** | **59.2** | **29** | **30** |
| **Control** | **52.6** | **53.6** | **28** | **27** |
| **Control** | **35.9** | **43.1** | **28** | **28** |
| **Control** | **54.4** | **53.5** | **28** | **27** |
| **Control** | **49.4** | **52.1** | **28** | **28** |
| **Control** | **53.9** | **51.3** | **25** | **28** |
| **Control** | **43.7** | **45.2** | **27** | **29** |
| **Control** | **57.6** | **60.4** | **29** | **28** |
| **Control** | **63.8** | **66.3** | **29** | **28** |
| **Control** | **52.9** | **53.4** | **30** | **28** |
| **Control** | **60.1** | **64.7** | **30** | **28** |
| **Control** | **48.4** | **43.3** | **29** | **26** |
| **Control** | **73.9** | **74.5** | **29** | **29** |
| **Control** | **64.8** | **62.0** | **30** | **30** |
| **Control** | **45.5** | **55.0** | **28** | **28** |
| **Control** | **57.2** | **55.0** | **28** | **28** |
| **Control** | **67.2** | **56.9** | **29** | **28** |
| **Control** | **63.3** | **64.0** | **28** | **26** |
| **Control** | **79.7** | **78.1** | **30** | **30** |
| **Control** | **52.8** | **50.4** | **29** | **30** |
| **Control** | **60.0** | **58.7** | **30** | **28** |
| **Control** | **48.8** | **50.3** | **29** | **28** |
| **Control** | **51.7** | **57.4** | **29** | **30** |
| **Control** | **47.6** |  | **26** | **29** |
| **Control** | **48.1** | **55.6** | **29** | **27** |
| **Patient** | **32.5** | **32.6** | **28** | **22** |
| **Patient** | **44.1** | **42.0** | **23** | **21** |
| **Patient** | **50.9** |  | **27** | **23** |
| **Patient** | **41.5** | **41.7** | **22** | **20** |
| **Patient** | **40.2** | **44.9** | **27** | **24** |
| **Patient** | **39.8** |  | **24** | **22** |
| **Patient** | **46.1** | **47.5** | **22** | **19** |
| **Patient** | **42.4** | **33.5** | **27** | **21** |
| **Patient** | **37.4** | **37.1** | **26** | **23** |
| **Patient** | **41.0** | **42.3** | **28** | **24** |
| **Patient** | **50.6** | **54.1** | **21** | **20** |
| **Patient** | **58.6** | **56.4** | **29** | **24** |
| **Patient** | **46.2** | **49.4** | **25** | **23** |
| **Patient** | **43.4** | **47.5** | **24** | **20** |
| **Patient** | **37.6** | **36.6** | **26** | **24** |
| **Patient** | **39.3** | **36.2** | **26** | **18** |
| **Patient** | **44.8** | **40.4** | **23** | **20** |
| **Patient** | **47.3** | **54.2** | **26** | **24** |
| **Patient** | **48.2** | **48.9** | **25** | **23** |
| **Patient** | **40.0** | **38.6** | **24** | **19** |
| **Patient** | **42.3** |  | **23** | **21** |
| **Patient** | **39.7** | **38.4** | **27** | **18** |
| **Patient** | **37.6** | **35.4** | **24** | **19** |
| **Patient** | **46.7** | **45.0** | **23** | **19** |
| **Patient** | **44.7** | **50.9** | **23** | **18** |
| **Patient** | **32.8** |  | **21** | **13** |
| **Patient** | **39.1** |  | **25** | **20** |
| **Patient** | **47.9** | **50.8** | **21** | **19** |
