# Supplementary material for: Validation of a web-based self-administered test for cognitive assessment in a Swedish geriatric setting
Source: PLoS One. 2024 Feb 1;19(2):e0297575. doi: 10.1371/journal.pone.0297575 (PMC10833583; doi:10.1371/journal.pone.0297575)
Supplement: S5 Table — Number of correct answers for each BoT subtest. (DOCX) [file pone.0297575.s006.docx]

**S5 Table. Raw score.** Number of correct answers for each BoT subtest.

|  | **Control group** | | | | | | **Patient group** | | | | | |
| --- | --- | --- | --- | --- | --- | --- | --- | --- | --- | --- | --- | --- |
|  | First BoT-test | | | Second BoT-test | | | First BoT-test | | | Second BoT-test | | |
| **Subtest** | n | mean | SD | n | mean | SD | n | mean | SD | n | mean | SD |
| Attention task III | 30 | 8.2 | 2.5 | 28 | 8.6 | 2.3 | 28 | 5.8 | 2.5 | 23 | 6.0 | 2.3 |
| Visual memory task II | 29 | 7.9 | 3.2 | 28 | 7.6 | 2.7 | 24 | 3.3 | 3.1 | 20 | 4.2 | 3.5 |
| Delayed verbal memory task | 30 | 16.6 | 2.2 | 28 | 17.0 | 2.7 | 28 | 12.4 | 2.7 | 23 | 12.8 | 2.0 |
| Calculus task | 30 | 12.7 | 5.4 | 28 | 13.1 | 3.6 | 27 | 6.3 | 5.4 | 23 | 6.4 | 4.6 |
| Color interference task | 30 | 16.6 | 5.0 | 28 | 17.8 | 4.5 | 27 | 12.1 | 2.4 | 23 | 11.7 | 4.7 |
| Verbal memory task II | 30 | 7.2 | 2.2 | 28 | 7.4 | 2.2 | 27 | 5.5 | 2.1 | 23 | 5.8 | 2.4 |
| Opposite task | 30 | 50.7 | 18.6 | 28 | 48.6 | 17.7 | 27 | 33.2 | 13.1 | 23 | 34.6 | 17.3 |
| Written comprehension | 30 | 10.8 | 4.2 | 28 | 11.0 | 4.3 | 27 | 7.4 | 2.5 | 23 | 7.3 | 3.2 |
| Word categories | 30 | 19.2 | 6.7 | 28 | 19.3 | 6.2 | 21 | 11.1 | 5.9 | 20 | 11.8 | 6.9 |
| Sequences | 27 | 11.5 | 6.5 | 28 | 11.6 | 6.7 | 24 | 5.3 | 3.9 | 21 | 5.6 | 3.9 |
| Puzzles | 29 | 4.2 | 2.4 | 28 | 4.2 | 1.9 | 19 | 2.2 | 1.3 | 21 | 2.7 | 1.6 |
